# Pediatric Resident Communication of Hospital Discharge Instructions

**DOI:** 10.3928/24748307-20230918-01

**Published:** 2023-10

**Authors:** Alexander F. Glick, Jonathan S. Farkas, Jasmine Gadhavi, Alan L. Mendelsohn, Nicole Schulick, H. Shonna Yin

## Abstract

**Objective:**

Suboptimal provider-parent communication contributes to poor parent comprehension of pediatric discharge instructions, which can lead to adverse outcomes. Residency is a critical window to acquire and learn to utilize key communication skills, potentially supported by formal training programs or visual reminders. Few studies have examined resident counseling practices or predictors of counseling quality. Our objectives were to (1) examine pediatric resident counseling practices and (2) determine how formal training and presence of discharge templates with domain-specific prompts are associated with counseling.

**Methods:**

We conducted a cross-sectional survey of a convenience sample of residents in the American Academy of Pediatrics Section on Pediatric Trainees. Outcomes included resident self-report of frequency of (1) counseling in domains of care and (2) use of health literacy-informed counseling strategies (pictures, demonstration, Teach Back, Show Back) (6-point scales; frequent = *often/usually/always*). Predictor variables were (1) formal discharge-related training (e.g., lectures) and (2) hospital discharge instruction template with space for individual domains. Logistic regression analyses, utilizing generalized estimating equations when appropriate to account for multiple domains (adjusting for resident gender, postgraduate year), were performed.

**Key Results:**

Few residents (*N* = 317) (13.9%) reported formal training. Over 25% of residents infrequently counsel on side effects, diagnosis, and restrictions. Resident reported use of communication strategies was infrequent: drawing pictures (24.1%), demonstration (15.8%), Teach Back (36.8%), Show Back (11.4%). Designated spaces in instruction templates for individual domains were associated with frequent domain-specific counseling (adjusted odds ratio [aOR] 4.1 [95% confidence interval: 3.5–4.8]). Formal training was associated with frequent Teach Back (aOR 2.6 [1.4–5.1]) and Show Back (aOR 2.7 [1.2–6.2]).

**Conclusions:**

Lack of formal training and designated space for domain-specific instructions are associated with suboptimal counseling at discharge by pediatric residents. Future research should focus on determining the best mechanisms for teaching trainees communication skills and optimizing written instruction templates to support verbal counseling. [***HLRP: Health Literacy Research and Practice*. 2023;7(4):e178–e186.**]

## Abstract (plain language summary)

**Plain Language Summary: **Pediatric residents infrequently counsel about several important domains of care or use health literacy-informed counseling strategies. Resident receipt of formal training in communication practices and presence of written instruction templates are associated with higher quality counseling. Future research is needed to determine how to better support trainees in learning how to provide high quality and effective communication with parents.

Optimizing transitions of care at hospital discharge has become a national priority (Auger et al., 2015; Berry et al., 2014). Approximately 15% of children are readmitted or have an emergency department or urgent care visit within 30 days of inpatient discharge (Auger et al., 2018); up to 30% of pediatric readmissions may be preventable (Toomey et al., 2016).

Suboptimal provider-patient/family communication is a central contributor to adverse outcomes post-discharge (Auger et al., 2015; Berry et al., 2014). Written patient instructions are often at a high reading grade level, lack key information, and are designed poorly (Rodriguez et al., 2022; Unaka et al., 2017; Yin et al., 2013). Provider verbal counseling is often missing essential content or uses medical jargon (Clark et al., 2005; Vashi & Rhodes, 2011). In addition, providers infrequently use health literacy-informed communication strategies, such as drawing pictures, demonstrating how to follow an instruction, having patients report back in their own words how they would perform a task (Teach Back), or asking individuals to demonstrate how they would follow an instruction (Show Back) (Turner et al., 2009; Yin et al., 2014). Consequently, parents make errors in comprehension of and adherence to discharge instructions, which cover numerous domains of care including medications, appointments, return precautions, and restrictions (Glick et al., 2017, 2019).

One barrier to use best practices for communication may be a lack of resident training. While the Accreditation Council for Graduate Medical Education (ACGME) expects trainees to receive education in communication surrounding transitions of care (Accreditation Council for Graduate Medical Education, 2020), few residents receive training. For example, less than half of emergency medicine residency programs (Gallahue et al., 2015) and only 16% of internal medicine residency programs (Aiyer et al., 2009) offer formal discharge-related training, such as specific lectures. Limited research has assessed the extent to which pediatric residents have been trained in discharge communication or how training is associated with communication practices at discharge.

Several other barriers, including a lack of structured and standardized written instructions, may also contribute to poor communication and subsequent parent errors in discharge plan management. Structured instructions are associated with improved comprehension and adherence (Ducharme et al., 2011; Waisman et al., 2005) and can enhance provider counseling (Yin et al., 2016), but few studies have examined how the use of structured written instructions affects pediatric discharge counseling. Additional barriers to suboptimal transitions include poor communication among team members and with families, low parent health literacy, language, and time (Auger et al., 2015; Okoniewska et al., 2015; Wong et al., 2011). Provider perspectives on barriers impacting care transitions have not been well studied.

Our primary objectives were (1) to examine pediatric resident counseling practices, including instruction on specific domains of care and use of counseling strategies, and (2) to determine how formal training in discharge-related counseling and the presence of standardized templates with domain-specific prompts are associated with counseling practices. Our secondary objective was to determine which barriers pediatric residents perceive to have the greatest impact on their ability to provide optimal discharge education.

## Methods

### Study Design and Participants

This was a cross-sectional study, which utilized an online survey tool (Qualtrics Software, Provo, UT). The survey **(Table A)** was designed based on interviews with residents and faculty and a review of the literature, ensuring inclusion of prioritized topics such as counseling in key domains of care (Albrecht et al., 2014; Horwitz et al., 2013) and health literacy-informed communication strategies (Yin et al., 2014). The survey was piloted with residents using cognitive interviews and modified prior to recruitment. Participants were recruited via an e-mail sent to members of the American Academy of Pediatrics (AAP) Section on Pediatric Trainees (SOPT [formerly the Section on Medical Students, Residents, and Fellowship Trainees]) listserv in June 2016. A reminder was sent 1 week later. SOPT had approximately 11,000 members at the time of survey distribution; all residents who are members of the AAP receive membership in SOPT. All postgraduate year (PGY), PGY-1, PGY-2, PGY-3, and PGY-4, residents were eligible. Consent was assumed by the participant's participation in the survey. This study was approved by the NYU Grossman School of Medicine Institutional Review Board and the SOPT executive committee.

**Table A:**
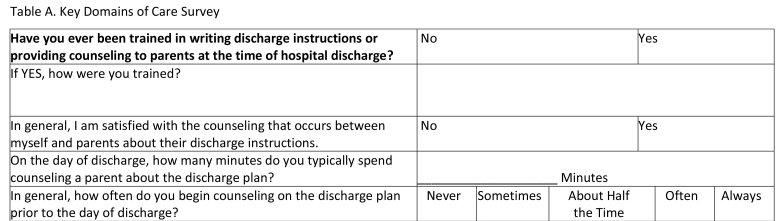
Key Domains of Care Survey

**Have you ever been trained in writing discharge instructions or providing counseling to parents at the time of hospital discharge?**	No			Yes		
If YES, how were you trained?						
In general, I am satisfied with the counseling that occurs between myself and parents about their discharge instructions.	No			Yes		
On the day of discharge, how many minutes do you typically spend counseling a parent about the discharge plan?	____________________ Minutes					
In general, how often do you begin counseling on the discharge plan prior to the day of discharge?	Never	Sometimes	About Half the Time		Often	Always

**Table:**
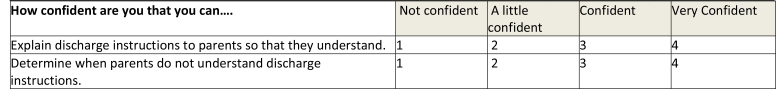


**How confident are you that you can….**	Not confident	A little confident	Confident	Very Confident
Explain discharge instructions to parents so that they understand.	1	2	3	4
Determine when parents do not understand discharge instructions.	1	2	3	4

**Table:**
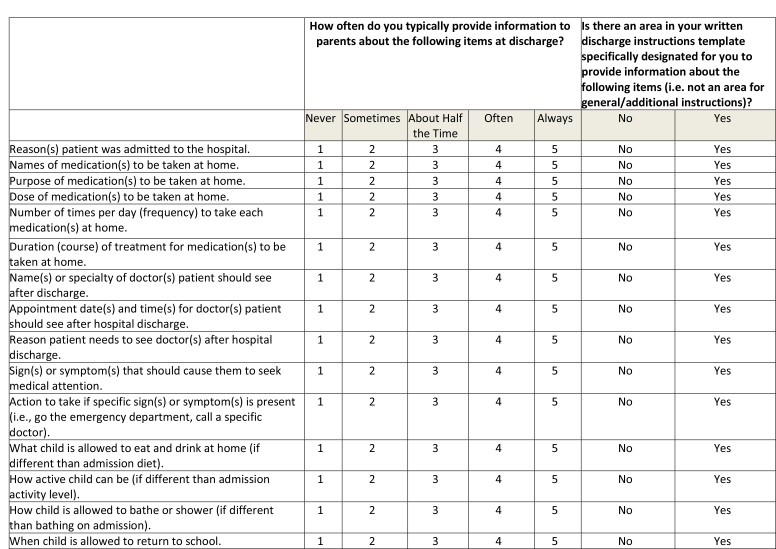


	**How often do you typically provide information to parents about the following items at discharge?**					**Is there an area in your written discharge instructions template specifically designated for you to provide information about the following items (i.e. not an area for general/additional instructions) ?**	
	Never	Sometimes	About Half the Time	Often	Always	No	Yes
Reason(s) patient was admitted to the hospital.	1	2	3	4	5	No	Yes
Names of medication(s) to be taken at home.	1	2	3	4	5	No	Yes
Purpose of medication(s) to be taken at home.	1	2	3	4	5	No	Yes
Dose of medication(s) to be taken at home.	1	2	3	4	5	No	Yes
Number of times per day (frequency) to take each medication(s) at home.	1	2	3	4	5	No	Yes
Duration (course) of treatment for medication(s) to be taken at home.	1	2	3	4	5	No	Yes
Name(s) or specialty of doctor(s) patient should see after discharge.	1	2	3	4	5	No	Yes
Appointment date(s) and time(s) for doctor(s) patient should see after hospital discharge.	1	2	3	4	5	No	Yes
Reason patient needs to see doctor(s) after hospital discharge.	1	2	3	4	5	No	Yes
Sign(s) or symptom(s) that should cause them to seek medical attention.	1	2	3	4	5	No	Yes
Action to take if specific sign(s) or symptom(s) is present (i.e., go the emergency department, call a specific doctor).	1	2	3	4	5	No	Yes
What child is allowed to eat and drink at home (if different than admission diet).	1	2	3	4	5	No	Yes
How active child can be (if different than admission activity level).	1	2	3	4	5	No	Yes
How child is allowed to bathe or shower (if different than bathing on admission).	1	2	3	4	5	No	Yes
When child is allowed to return to school.	1	2	3	4	5	No	Yes

**Table:**
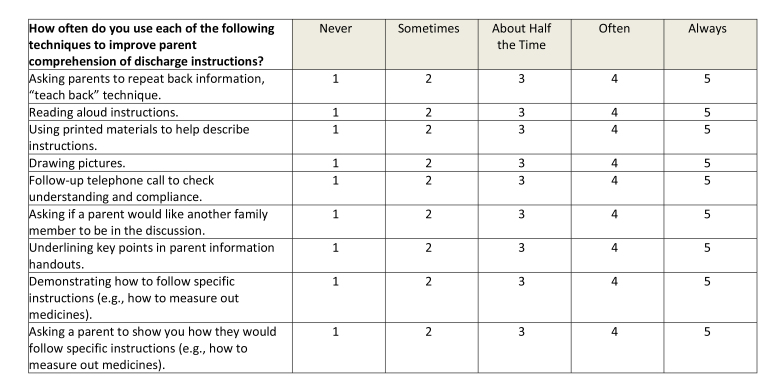


**How often do you use each of the following techniques to improve parent comprehension of discharge instructions?**	Never	Sometimes	About Half the Time	Often	Always
Asking parents to repeat back information, “teach back” technique.	1	2	3	4	5
Reading aloud instructions.	1	2	3	4	5
Using printed materials to help describe instructions.	1	2	3	4	5
Drawing pictures.	1	2	3	4	5
Follow-up telephone call to check understanding and compliance.	1	2	3	4	5
Asking if a parent would like another family member to be in the discussion.	1	2	3	4	5
Underlining key points in parent information handouts.	1	2	3	4	5
Demonstrating how to follow specific instructions (e.g., how to measure out medicines).	1	2	3	4	5
Asking a parent to show you how they would follow specific instructions (e.g., how to measure out medicines).	1	2	3	4	5

**Table:**
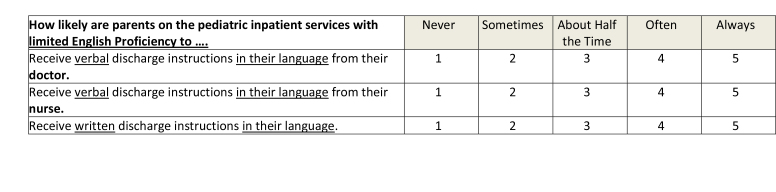


**How likely are parents on the pediatric inpatient services with limited English Proficiency to ….**	Never	Sometimes	About Half the Time	Often	Always
Receive verbal discharge instructions in their language from their **doctor.**	1	2	3	4	5
Receive verbal discharge instructions in their language from their **nurse.**	1	2	3	4	5
Receive written discharge instructions in their language.	1	2	3	4	5

**Table:**
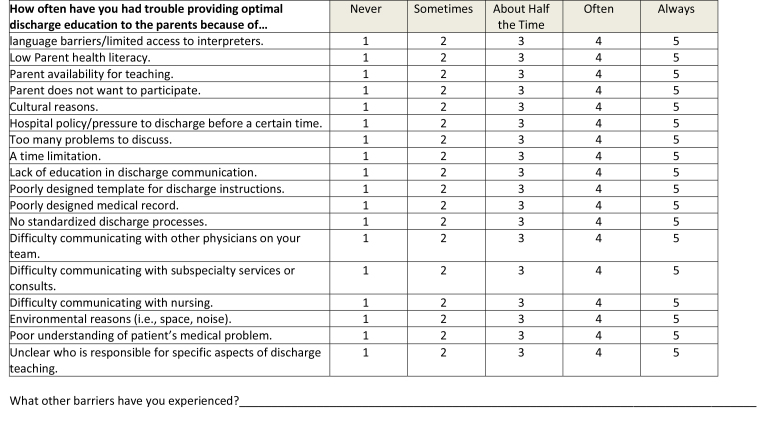


**How often have you had trouble providing optimal discharge education to the parents because of…**	Never	Sometimes	About Half the Time	Often	Always
language barriers/limited access to interpreters.	1	2	3	4	5
Low Parent health literacy.	1	2	3	4	5
Parent availability for teaching.	1	2	3	4	5
Parent does not want to participate.	1	2	3	4	5
Cultural reasons.	1	2	3	4	5
Hospital policy/pressure to discharge before a certain time.	1	2	3	4	5
Too many problems to discuss.	1	2	3	4	5
A time limitation.	1	2	3	4	5
Lack of education in discharge communication.	1	2	3	4	5
Poorly designed template for discharge instructions.	1	2	3	4	5
Poorly designed medical record.	1	2	3	4	5
No standardized discharge processes.	1	2	3	4	5
Difficulty communicating with other physicians on your team.	1	2	3	4	5
Difficulty communicating with subspecialty services or consults.	1	2	3	4	5
Difficulty communicating with nursing.	1	2	3	4	5
Environmental reasons (i.e., space, noise).	1	2	3	4	5
Poor understanding of patient's medical problem.	1	2	3	4	5
Unclear who is responsible for specific aspects of discharge teaching.	1	2	3	4	5
What other barriers have you experienced?________________________________________________________________________________

**Table:**
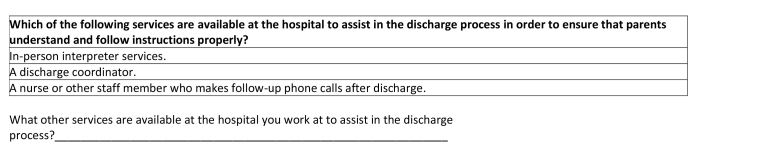


**Which of the following services are available at the hospital to assist in the discharge process in order to ensure that parents understand and follow instructions properly?**
In-person interpreter services.
A discharge coordinator.
A nurse or other staff member who makes follow-up phone calls after discharge.
What other services are available at the hospital you work at to assist in the discharge process?______________________________________________________________

**Table:**
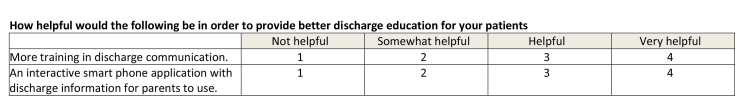


**How helpful would the following be in order to provide better di scharge education for your patients**				
	Not helpful	Somewhat helpful	Helpful	Very helpful
More training in discharge communication.	1	2	3	4
An interactive smart phone application with discharge information for parents to use.	1	2	3	4

**Table:**
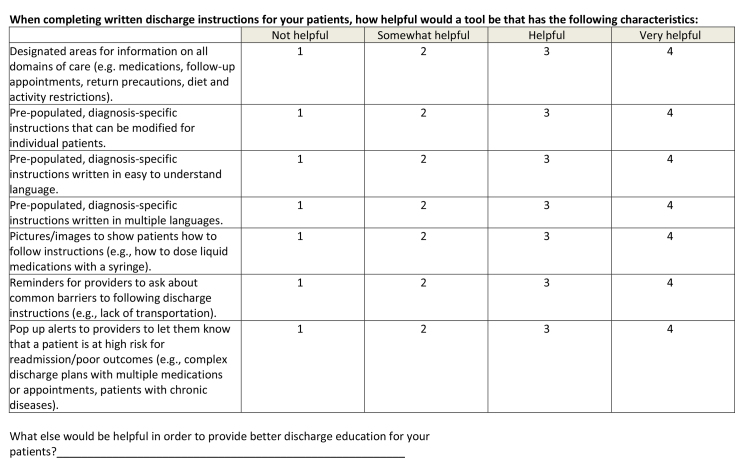


**When completing written discharge instructions for your patients, how helpful would a tool be that has the following characteristics:**				
	Not helpful	Somewhat helpful	Helpful	Very helpful
Designated areas for information on all domains of care (e.g. medications, follow-up appointments, return precautions, diet and activity restrictions).	1	2	3	4
Pre-populated, diagnosis-specific instructions that can be modified for individual patients.	1	2	3	4
Pre-populated, diagnosis-specific instructions written in easy to understand language.	1	2	3	4
Pre-populated, diagnosis-specific instructions written in multiple languages.	1	2	3	4
Pictures/images to show patients how to follow instructions (e.g., how to dose liquid medications with a syringe).	1	2	3	4
Reminders for providers to ask about common barriers to following discharge instructions (e.g., lack of transportation).	1	2	3	4
Pop up alerts to providers to let them know that a patient is at high risk for readmission/poor outcomes (e.g., complex discharge plans with multiple medications or appointments, patients with chronic diseases).	1	2	3	4
What else would be helpful in order to provide better discharge education for your patients?________________________________________________________

**Table:**
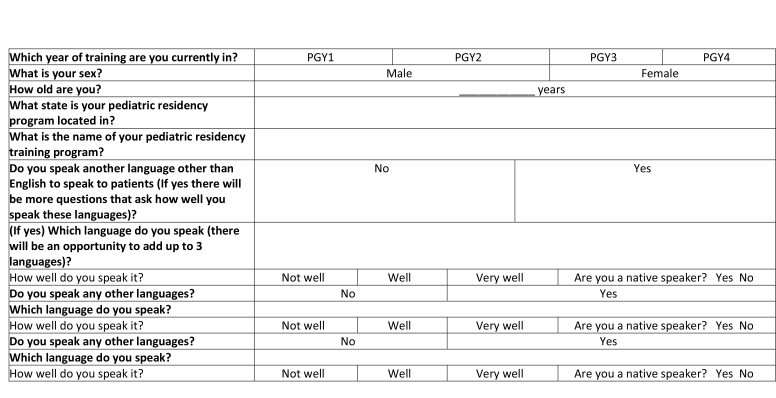


**Which year of training are you currently in?**	PGY1		PGY2	PGY3	PGY4
**What is your sex?**	Male			Female	
**How old are you?**	____________ years				
**What state is your pediatric residency program located in?**					
**What is the name of your pediatric residency training program?**					
**Do you speak another language other than English to speak to patients (If yes there will be more questions that ask how well you speak these languages)?**	No			Yes	
**(If yes) Which language do you speak (there will be an opportunity to add up to 3 languages)?**					
How well do you speak it?	Not well	Well	Very well	Are you a native speaker? Yes No	
**Do you speak any other languages?**	No		Yes		
**Which language do you speak?**					
How well do you speak it?	Not well	Well	Very well	Are you a native speaker? Yes No	
**Do you speak any other languages?**	No		Yes		
**Which language do you speak?**					
How well do you speak it?	Not well	Well	Very well	Are you a native speaker? Yes No	

### Primary Predictor Variables

***Training. ***We asked participants “Have you ever been trained in writing discharge instructions or providing counseling to parents at the time of hospital discharge?” (Yes/No). Those who had been trained were asked to provide a free-text description of their training. Two investigators (A.F.G. and J.S.F.) independently reviewed subject descriptions of training and categorized free-text responses as formal (e.g., lecture) or informal (e.g., feedback by senior resident) training; inter-rater reliability was strong (*κ* = 0.93). Disagreements were resolved by a consensus discussion.

***Designated space for domain-specific instructions.*** We asked participants if there is an area in their written discharge instructions template specifically designated for information about key domains of care **(Table A)**. To capture the extent to which there exist discharge instruction templates that cover all key domains, a composite variable was created.

### Primary Outcome Variable: Counseling Practices in Individual Domains of Care

To assess counseling practices, we asked participants about frequency of counseling in key domains. They rated their answers on a 6-point Likert scale (*never, seldom, sometimes, often, usually, always*). We dichotomized answers into those who frequently (*often, usually, always*) and infrequently (*never, seldom, sometimes*) provided counseling in these domains. A composite variable, which examined whether residents frequently counseled in all domains of care, was created.

### Primary Outcome Variable: Use of Advanced Counseling Strategies

We asked participants about frequency (6-point Likert scale) of use of drawing pictures, demonstration, Teach Back, and Show Back. We dichotomized answers into those who frequently (*often, usually, always*) and infrequently (*never, seldom, sometimes*) used these strategies.

### Secondary Outcome Variable: Perceived Barriers

We asked participants about frequency of specific barriers (e.g., parent low health literacy, time, language) that they perceived might affect discharge counseling. Answers reported on a 6-point Likert scale were dichotomized into frequent and infrequent as described for use of counseling strategies.

### Demographics

We assessed resident post-graduate level (PGY-1, 2, 3, or 4) and gender.

### Data Analysis

We used descriptive statistics to assess all primary and secondary outcomes.

For bivariate analyses, we used Fisher's exact test and Chi-square test to evaluate whether formal training or the presence of designated space in discharge instructions for a given domain were associated with frequent resident counseling in these individual domains. We also examined whether formal training and a presence of a designated space for all domains (composite variable) were associated with frequent counseling in all domains (composite variable). We assessed associations between the primary predictor variable of formal training and use of individual counseling strategies.

To assess predictors of frequent counseling in individual domains of care in a single adjusted analysis, we utilized generalized estimating equations (exchangeable correlation structure, binomial distribution, logit link). Predictor variables included (1) designated space in the discharge plan template for individual domains and (2) resident report of formal training. We adjusted for resident post-graduate level and gender given prior associations with counseling practices (Henderson & Weisman, 2001; Krugman et al., 2009).

For cases in which formal training was associated with individual counseling strategies in bivariate analyses, we also performed logistic regressions, adjusting for resident post-graduate level, gender, and a composite variable of presence of a designated space for all domains of care.

A *p* value *of* < .05 was considered statistically significant. Analyses were performed using Stata SE 12.1 (StataCorp, College Station, TX).

## Results

### Sample Characteristics

A total of 465 pediatric residents started the electronic survey. The final analysis included 317 residents who completed all questions related to primary predictor and outcome variables. Residents came from 140 programs; 52 residents did not report their residency program. Most respondents were women; about three-quarters were in their first 2 years of residency (**Table 1**).

**Table 1:**
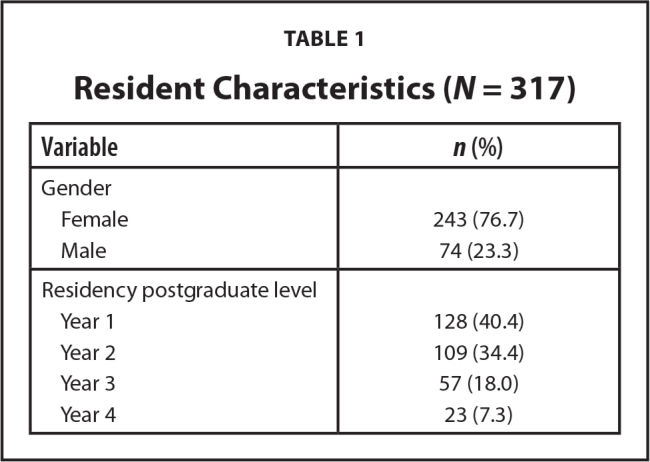
Resident Characteristics (*N* = 317)

**Variable**	***n* (%)**

Gender	
Female	243 (76.7)
Male	74 (23.3)

Residency postgraduate level	
Year 1	128 (40.4)
Year 2	109 (34.4)
Year 3	57 (18.0)
Year 4	23 (7.3)

### Resident Counseling Practices

Over 90% reported frequently providing counseling on the medications (name, reason, duration), return precautions, and which physicians to see after discharge (**Table 2**). Approximately 40% of residents reported frequently counseling parents on medication side effects. Overall, 21.8% of residents reported provision of frequent counseling in all domains of care.

**Table 2:**
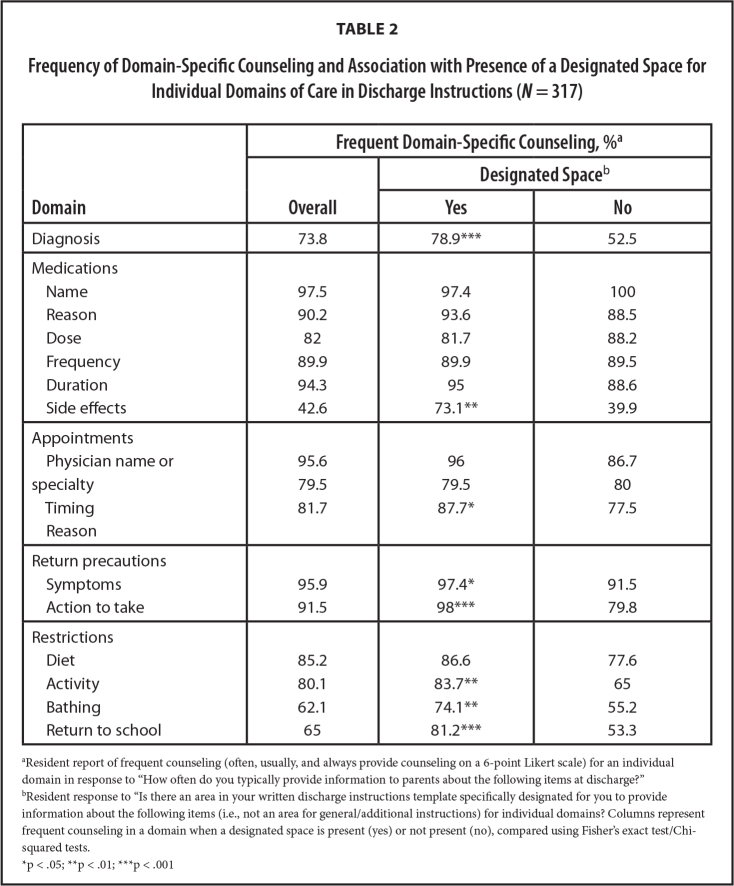
Frequency of Domain-Specific Counseling and Association with Presence of a Designated Space for Individual Domains of Care in Discharge Instructions (*N* = 317)

**Domain**	**Frequent Domain-Specific Counseling, %^a^**		

	**Overall**	**Designated Space** ^b^	

		**Yes**	**No**

Diagnosis	73.8	78.9^***^	52.5

Medications			
Name	97.5	97.4	100
Reason	90.2	93.6	88.5
Dose	82	81.7	88.2
Frequency	89.9	89.9	89.5
Duration	94.3	95	88.6
Side effects	42.6	73.1^**^	39.9

Appointments			
Physician name or	95.6	96	86.7
specialty	79.5	79.5	80
Timing	81.7	87.7^*^	77.5
Reason			

Return precautions			
Symptoms	95.9	97.4^*^	91.5
Action to take	91.5	98^***^	79.8

Restrictions			
Diet	85.2	86.6	77.6
Activity	80.1	83.7^**^	65
Bathing	62.1	74.1^**^	55.2
Return to school	65	81.2^***^	53.3

a Resident report of frequent counseling (often, usually, and always provide counseling on a 6-point Likert scale) for an individual domain in response to “How often do you typically provide information to parents about the following items at discharge?”

b Resident response to “Is there an area in your written discharge instructions template specifically designated for you to provide information about the following items (i.e., not an area for general/additional instructions) for individual domains? Columns represent frequent counseling in a domain when a designated space is present (yes) or not present (no), compared using Fisher's exact test/Chi-squared tests.

* p < .05;

** p < .01;

*** p < .001

Approximately one-third (36.8%) of residents reported frequently using Teach Back. Fewer residents reported that they frequently draw pictures (24.1%), demonstrate how to follow instructions (15.8%), or utilize Show Back (11.4%) during discharge counseling.

### Training in Discharge Education

Around half of respondents reported having ever being trained in writing discharge instructions or providing verbal discharge counseling. Participants reported a variety of different types of training, ranging from formal lectures to senior residents providing informal guidance; 13.9% of respondents received formal training.

### Designated Space in Discharge Plans for Individual Domains

Over 90% reported that their discharge plan template has a designated area to document instructions related to the medication name, dose, and frequency, as well information on the physician's name and specialty and on timing of appointments. Fewer than 10% noted that their templates have a specified space for medication side effects. Less than 50% reported having discharge plans with designated spaces for the diagnosis, reason for a child's medications or appointments, bathing restrictions, or when to return to school. Approximately 5% reported that their institution's discharge plan templates had designated spaces for all domains asked about on the survey.

### Variables Associated with Domain-Specific Counseling

There were no significant associations between formal training and resident report of frequent counseling in any individual domains asked about. Resident report of formal training was associated with report of frequent counseling in all domains of care (composite variable) (34.1 vs. 19.8%, *p* = .047).

Presence of a designated space in the discharge plan template for domain-specific instructions was associated with frequent provider counseling in that domain for diagnosis, medication side effects, reason for appointments, return precautions (symptoms to watch for and action to take), and restrictions (diet, activity, bathing, and return to school) (**Table 2**). A composite variable indicating presence of designated spaces for all domains was associated with a composite variable indicating counseling in all domains of care (53.3 vs. 20.2%, *p* = .006).

In adjusted analyses, designated spaces in the discharge plan template for individual domains (adjusted odds ratio [aOR] 4.1, 95% confidence interval [CI] 3.5–4.8, *p* < .001) and being a woman resident (aOR 1.6, 95% CI 1.2–2.2, *p* = .001) were significantly associated with frequent counseling in that domain. Formal discharge-related training was not associated with frequent domain-specific counseling (aOR 1.4, 95% CI 0.9–2.1, *p* = .09; **Table 3**).

**Table 3:**
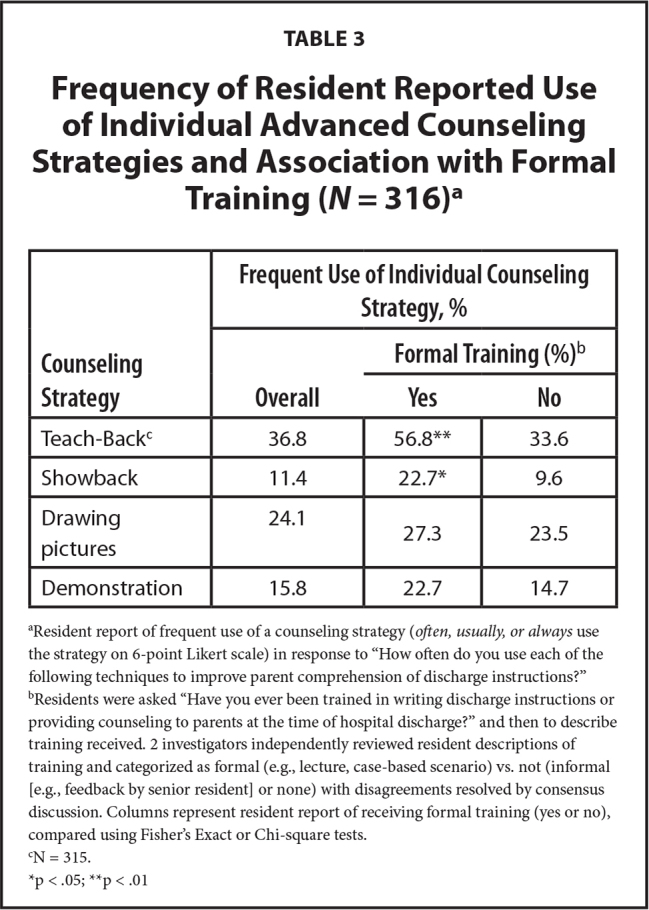
Frequency of Resident Reported Use of Individual Advanced Counseling Strategies and Association with Formal Training (*N* = 316)^a^

**Counseling Strategy**	**Frequent Use of Individual Counseling Strategy, %**		
	**Overall**	**Formal Training (%)** ^b^	
		**Yes**	**No**
Teach-Back^c^	36.8	56.8^**^	33.6
Showback	11.4	22.7^*^	9.6
Drawing pictures	24.1	27.3	23.5
Demonstration	15.8	22.7	14.7

a Resident report of frequent use of a counseling strategy (*often, usually, or always *use the strategy on 6-point Likert scale) in response to “How often do you use each of the following techniques to improve parent comprehension of discharge instructions?”

b Residents were asked “Have you ever been trained in writing discharge instructions or providing counseling to parents at the time of hospital discharge?” and then to describe training received. 2 investigators independently reviewed resident descriptions of training and categorized as formal (e.g., lecture, case-based scenario) vs. not (informal [e.g., feedback by senior resident] or none) with disagreements resolved by consensus discussion. Columns represent resident report of receiving formal training (yes or no), compared using Fisher's Exact or Chi-square tests.

c N = 315.

* p < .05;

** p < .01

### Variables Associated with Use of Advanced Counseling Strategies

In bivariate analyses, formal training was associated with frequent use of Teach Back (56.8 vs. 33.6%, *p* = .004) and Show Back (22.7 vs. 9.6%, *p* = .02). Formal training was not associated with frequent use of drawing pictures or demonstration (**Table 4**).

**Table 4:**
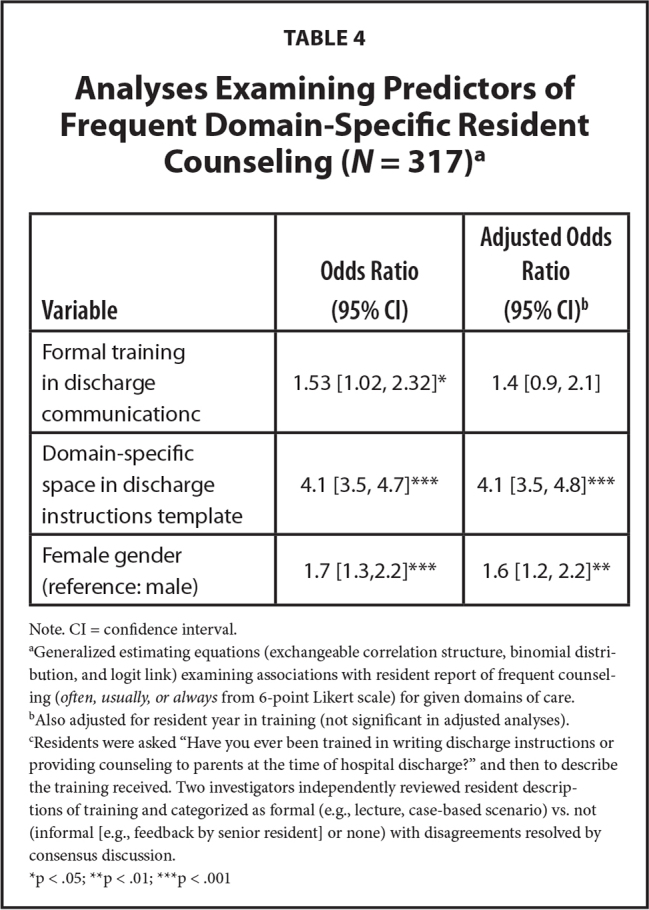
Analyses Examining Predictors of Frequent Domain-Specific Resident Counseling (*N* = 317)^a^

**Variable**	**Odds Ratio (95% CI)**	**Adjusted Odds Ratio (95% CI)^b^**
Formal training in discharge communication^c^	1.53 [1.02, 2.32]^*^	1.4 [0.9, 2.1]
Domain-specific space in discharge instructions template	4.1 [3.5, 4.7]^***^	4.1 [3.5, 4.8]^***^
Female gender (reference: male)	1.7 [1.3,2.2]^***^	1.6 [1.2, 2.2]^**^

Note. CI = confidence interval.

a Generalized estimating equations (exchangeable correlation structure, binomial distribution, and logit link) examining associations with resident report of frequent counseling (*often, usually, or always* from 6-point Likert scale) for given domains of care.

b Also adjusted for resident year in training (not significant in adjusted analyses).

c Residents were asked “Have you ever been trained in writing discharge instructions or providing counseling to parents at the time of hospital discharge?” and then to describe the training received. Two investigators independently reviewed resident descriptions of training and categorized as formal (e.g., lecture, case-based scenario) vs. not (informal [e.g., feedback by senior resident] or none) with disagreements resolved by consensus discussion.

* p < .05;

** p < .01;

*** p < .001

In adjusted analyses, formal training was associated with frequent use of both Teach Back (aOR 2.6, 95% CI 1.4–5.1, *p* = .004) and Show Back (aOR 2.7 [95% CI 1.2–6.2], *p* = .02).

### Perceived Barriers

Participants noted several barriers perceived to affect their ability to provide optimal discharge education (**Table 5**). One-half of the participants reported low health literacy in parents as a barrier. A lack of time was a notable barrier for more than 40% of participants, while nearly 30% perceived language to be a barrier.

**Table 5:**
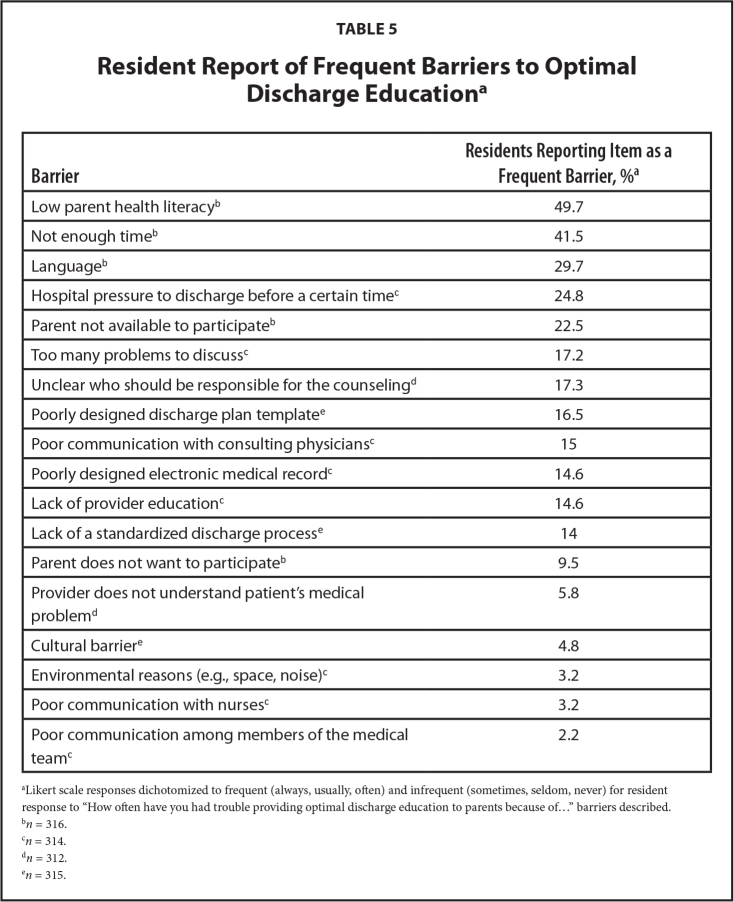
Resident Report of Frequent Barriers to Optimal Discharge Education^a^

**Barrier**	**Residents Reporting Item as a Frequent Barrier, %^a^**
Low parent health literacy^b^	49.7
Not enough time^b^	41.5
Language^b^	29.7
Hospital pressure to discharge before a certain time^c^	24.8
Parent not available to participate^b^	22.5
Too many problems to discuss^c^	17.2
Unclear who should be responsible for the counseling^d^	17.3
Poorly designed discharge plan template^e^	16.5
Poor communication with consulting physicians^c^	15
Poorly designed electronic medical record^c^	14.6
Lack of provider education^c^	14.6
Lack of a standardized discharge process^e^	14
Parent does not want to participate^b^	9.5
Provider does not understand patient's medical problem^d^	5.8
Cultural barrier^e^	4.8
Environmental reasons (e.g., space, noise)^c^	3.2
Poor communication with nurses^c^	3.2
Poor communication among members of the medical team^c^	2.2

a Likert scale responses dichotomized to frequent (always, usually, often) and infrequent (sometimes, seldom, never) for resident response to “How often have you had trouble providing optimal discharge education to parents because of…” barriers described.

b *n* = 316.

c *n* = 314.

d *n* = 312.

e *n* = 315.

## Discussion

This cross-sectional survey found that pediatric residents reported infrequent counseling across multiple domains of care, as well as infrequent use of recommended counseling strategies at hospital discharge. Formal training in discharge communication, reported by only 13.9% of residents, was associated with domain-specific counseling and use of Teach Back and Show Back. Designated spaces in instruction templates for individual domains were associated with frequent domain-specific counseling.

We identified several areas in which residents do not routinely counsel parents at discharge. Notably, fewer than one-half of participants typically provide information to parents on side effects, confirming low rates of side effects counseling seen in other studies (Olson & Windish, 2010) and which may explain findings that parents have poor comprehension of side effects (Glick et al., 2019). Given that counseling on side effects is associated with lower rates of adverse drug events (Forster et al., 2005), side effects should be one important area to focus on as part of routine counseling.

While we found that 96% of residents reported routinely discussing return precautions, or the concerning signs and symptoms to monitor for, prior work found that residents do not feel confident in their ability to educate in this area (Carnahan & Fletcher, 2015) and that parents often have difficulty comprehending information on return precautions (Glick et al., 2017, 2019). One prior survey of emergency medicine residency programs found that about 25% of discharge conversations do not include a discussion of return precautions and other key aspects of the discharge plan (Gallahue et al., 2015). It is possible that residents may be providing incomplete counseling related to return precautions. Future studies should examine the quality of instructions related to return precautions and their impact on parent comprehension and clinical outcomes.

Our study also found that few residents routinely use recommended health literacy-informed communication strategies during discharge counseling. One third of residents reported using Teach Back frequently. Other strategies were used frequently by less than one quarter of residents, consistent with prior studies that found infrequent providers use of these strategies (Turner et al., 2009; Yin et al., 2014). While one would not expect these strategies to be used in every encounter, use of these communication techniques can lead to improved outcomes, such as higher comprehension of provider instructions (Griffey et al., 2015) and improved glycemic control for patients with diabetes (Schillinger et al., 2003). Future work should examine how to best incorporate these strategies into routine practice.

We found that few pediatric residents receive formal training in discharge communication, consistent with limited training reported in other disciplines (Aiyer et al., 2009; Gallahue et al., 2015). Formal training was associated with higher use of Teach Back and Show Back, suggesting that residents who received formal training may provide higher quality counseling. A variety of members of the healthcare team (e.g., interns, residents, attendings, nurses) provide discharge counseling, although specific roles vary among institutions and sometimes are not well delineated (Ashbrook et al., 2013; Trivedi et al., 2021). The American Board of Pediatrics and ACGME expect residents to be adept at providing education to families at discharge and other transitions of care (Accreditation Council for Graduate Medical Education, 2020; The American Board of Pediatrics, 2021). The expectations that residents will perform discharge counseling, our study's findings, and the fact providers want additional training in discharge communication (Aiyer et al., 2009; Carnahan & Fletcher, 2015) support recommendations from the ACGME that residents should receive additional education in communication related to care transitions (Accreditation Council for Graduate Medical Education, 2014).

We also found that structured discharge instruction templates, or presence of a designated area in the template for a given domain of care, was associated with counseling in that particular domain. This is not surprising given that standardized instructions are associated with better comprehension of and adherence to instructions (Ducharme et al., 2011; Waisman et al., 2005) and higher quality provider counseling (Yin et al., 2016). These structured discharge instruction templates may serve as prompts for clinicians to provide comprehensive education. Such templates might support residents in developing habits for being more routine about providing a more comprehensive approach to counseling families and then continuing such practices when they become independent practitioners. It is important, however, to balance such structured formats while also keeping education patient- and family-centered (Auger et al., 2015; Berry et al., 2014).

Residents identified several barriers to optimal discharge education in our survey. Half of the respondents believed that low parent health literacy was a frequent barrier. This is not surprising as one third of parents in the United States have limited health literacy (Yin et al., 2009). Furthermore, nearly one third of residents reported that language affected discharge communication; this aligns with prior studies showing that language barriers in provider-parent communication are associated with poor parent comprehension of instructions, non-adherence, and adverse events (Khan et al., 2020; Samuels-Kalow et al., 2013). Incorporating formal training in health literacy-informed communication strategies as part of residency training and improving access to language services (e.g., interpreters, written translation services) may be beneficial. Other important barriers included lack of provider time and hospital pressure to discharge patients before a certain time. These barriers should be studied further to inform the design of interventions that not only incorporate evidence-based communication strategies, but also fit into hospital and provider workflow.

## Study Limitations

Our survey utilized a convenience sample of the SOPT listserv, which may have led to selection bias (e.g., selecting only those with the strongest opinions on the discharge process). In addition, while there were 11,000 reported members of the listserv and only 317 members who completed the survey, there is no way of knowing exactly how many of these were active members as resident AAP members receive automatic membership in this group (American Academy of Pediatrics, 2022). The study was also limited by self-reported data with no objective measures of discharge counseling or type of training received. Due to social desirability bias, participants may have overestimated how frequently they provide counseling on given domains of care, so counseling rates may actually be lower. The quality of counseling was also not assessed. Finally, the survey provided specific examples of barriers to the discharge process, and it is unclear which barriers residents would have identified without prompting.

## Conclusions

Our study suggests that residents may benefit from more standardized training on how to effectively communicate with parents at discharge, aligning with ACGME recommendations. Future work should focus on the design of training programs that will lead to improved resident counseling ability, and subsequent improved parent comprehension of and adherence to discharge instructions. In addition, future studies should examine the impact of more structured discharge instruction templates, as well as understanding more about how the entire inpatient team, including attendings and nurses, can work together to provide optimal discharge counseling.
